# Fatal invasive pulmonary aspergillosis in non-immunocompromised host: A case report

**DOI:** 10.1097/MD.0000000000035702

**Published:** 2023-10-27

**Authors:** Wanping Ao, Ping Huang, Jinjing Wang, Xiaoyun Fu, Bao Fu

**Affiliations:** a Department of Critical Care Medicine, Affiliated Hospital of Zunyi Medical University, Zunyi city, China; b Department of Pathology, Affiliated Hospital of Zunyi Medical University, Zunyi city, China.

**Keywords:** combination therapy, invasive pulmonary aspergillosis, non-immunocompromised host, voriconazole

## Abstract

**Rationale:**

Invasive pulmonary aspergillosis (IPA) is an uncommon but life-threatening disease. The disease often occurs in immunocompromised patients or critically ill patients. Here, we reported that IPA occurred in a non-immunocompromised host.

**Patient concerns:**

A 45-year-old man was admitted to the hospital for 1 week due to fever and cough. He was engaged in waste recycling and lived in a dark and humid environment for a long time.

**Diagnosis:**

Invasive pulmonary aspergillosis.

**Interventions:**

Next generation sequencing and pathological examination of alveolar lavage fluid indicated aspergillus infection. He received voriconazole infusion after admission. After 5 weeks of antifungal treatment, his condition improved significantly and discharged.

**Outcome:**

One week after discharge, his condition deteriorated again and returned to the hospital. Unfortunately, he died.

**Lesson:**

The immunocompetent adults can develop invasive pulmonary aspergillosis if they are exposed to high-risk environments. IPA in non-immunocompromised host should arouse the vigilance of clinicians.

## 1. Introduction

Pulmonary aspergillosis refers to a series of diseases caused by Aspergillus fungus infecting the lungs. The invasive pulmonary aspergillosis (IPA) is a serious disease not only in patients with severe immune deficiency, but also in critically ill patients and patients with chronic obstructive pulmonary disease. However, IPA has also been reported in some non-immunocompromised patients.^[1]^ Here, we report a severe IPA in an adult patient with immunocompetent.

## 2. Case presentation

**Figure 1.**
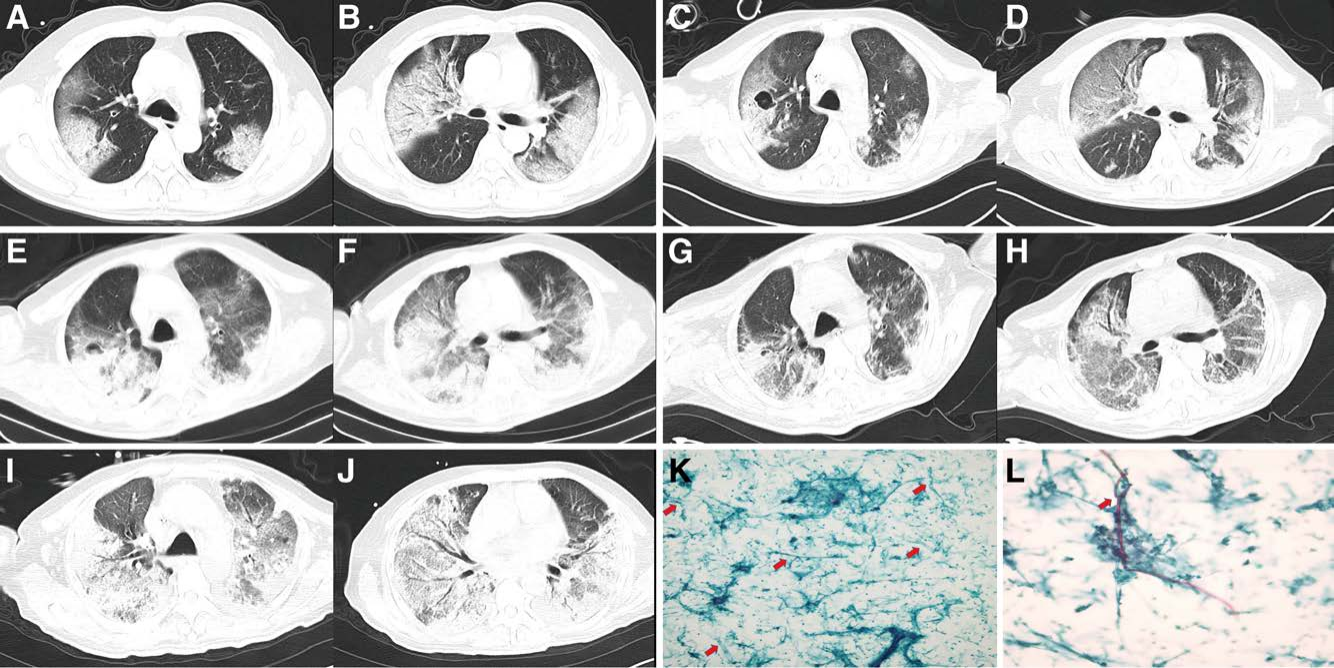
The evolution of chest CT during hospitalization and the histopathology of alveolar lavage fluid. (A and B) The CT at admission showed bilateral pneumonia and partial consolidation of the lung. (C and D) Two wk after admission, CT showed patchy shadows in both lungs and multiple cavities in the right lung. (E and F) CT scan at 3 weeks after admission showed that the left lung lesions decreased and the right lung lesions increased. (G and H) After 4 weeks, chest CT showed that the exudative lesions of both lungs decreased and the right pulmonary cavity narrowed or disappeared. (I and J) When the patient was hospitalized again, CT showed that both lung lesions increased significantly. (K and L) Microscopic examination of alveolar lavage fluid showed a large number of Aspergillus. Aspergillus shows dichotomous acute angle branching septate hyphae. CT = computed tomography.

A 45-year-old male was admitted to hospital for coughing 7 days. The patient was in good health before admission. He has been engaged in waste recycling for 3 years. Physical examination showed that T 36.6°C heart rate 115/min; respiratory rate 30/min; blood pressure 142/107mmHg; SP0_2_ 80% (air). Laboratory testing showed white blood cell 3.38 × 10^9^/L (normal range: 4–10 × 10^9^/L), neutrocyte proportion 0.80 (normal range: 0.45–0.75) and lymphocyte proportion 0.16 (normal range: 0.20–0.50). Other laboratory tests showed alanine aminotransferase 78U/L (normal range: 7–40) U/L, aspartate aminotransferase 278U/L (normal range: 7–40) U/L (normal range: 13–35) U/L, creatinine 147μmol/L (normal range: 30–90μmol/L), creatine kinase 3322 U/L(normal range: 38–174 U/L), creatine kinase isoenzyme 51 U/L(normal range: 0–24 U/L), hypersensitive C-reactive protein 27.27mg/L(normal range: 0.068–8.200 mg/L) and procalcitonin 1.5ng/mL (normal range: <0.5ng/mL). On admission, chest computed tomography (CT) showed bilateral lung pneumonia and consolidation of some areas (Fig. 1A and B).

After ICU admission, he received endotracheal intubation and prone ventilation. At the same time, he was given intravenous infusion of meropenem (1.0g, q8h) and voriconazole (250mg, q12h). After 16 hours of prone ventilation, his oxygenation index increased from 60mmHg to 120 mm Hg. Ventilator parameter setting: FiO2 100%, VT 370mL, RR 20/min, PEEP 14cmH_2_O. Subsequently, the next-generation sequencing (NGS) technologies of blood and sputum showed Aspergillus (Aspergillus fumigatus and Aspergillus flavus). Lymphocyte subsets showed CD4^+^ 376.0/μL (normal range: 414–1440/μL), CD8^+^ 228.0/μL (normal range: 238–1250/μL), CD3^+^ 640.0/μL (normal range: 770–2860/μL), CD4^+^/ CD8^+^ 1.65 (normal range: 0.7–2.87). Inflammatory factor level: Interleukin (IL)-2 5.38pg/mL (normal range:0.08–5.71 pg/mL), IL-4 5.91pg/mL (normal range:0.10–2.80 pg/mL), IL-6 78.51 pg/mL(normal range:1.18–5.30 pg/mL), IL-10 66.74pg/mL (normal range:0.19–4.91 pg/mL), TNF-α 0.66 pg/mL (normal range:0.10–2.31 pg/mL), IFN-γ 1.94 pg/mL (normal range:0.16–7.42 pg/mL). Serum (1,3)-beta-D-glucan, 4 virus (TORCH-IGM) examination and antinuclear antibody spectrum were negative. A large number of Aspergillus were found in the alveolar lavage fluid (Fig. 1K). Aspergillus shows bifurcating acute-angled branches and separating hyphae, as shown in Figure 1L.

After 1 week of ICU admission, the anti-infection regimen was adjusted to voriconazole 250mg q12h, caspofungin 50mg, piperacillin tazobactam 4.5g q12h. The patient fever disappeared. However, chest CT showed large areas of increased density and fuzzy shadow in both lungs, accompanied by multiple cavities in the right lung (Fig. 1C and D) at 2 weeks later. Three weeks later, chest CT showed that there were more exudative lesions in both lungs than the previous examination, and there were multiple cavities in the right lung (Fig. 1E and F). At the same time, he received tracheotomy. After 4 weeks, chest CT showed that the exudative lesions of both lungs decreased and the right pulmonary cavity narrowed or disappeared (Fig. 1G and H). The patient had no fever, no respiratory distress, and was successfully weaned. After 5 weeks, the patient tracheostomy cannula was successfully removed. The patient family asked to be discharged. Voriconazole 250mg was taken orally outside the hospital for q12h.

One week after discharge, the patient developed fever and respiratory distress again. Chest CT on admission showed an increase in bilateral lung lesions (Fig. 1I and K). He received immediate antifungal treatment with voriconazole (q12h). After admission, he received with endotracheal intubation and prone ventilation. However, 1 day later, the patient died of sudden cardiac arrest.

## 3. Discussion and conclusions

The study reports a case of invasive Aspergillus infection in a patient with normal immune function. Previous studies showed that 5% to 7% of IPA occur in non-immunocompromised patients.^[2]^ The pathophysiology of IPA in immunocompetent patients remains unclear. Among non-immunocompromised patients, patients with pulmonary disease have shown a tendency to develop aspergillosis, as demonstrated in some previous articles have reported cases of IPA with drowning, asthma or chronic obstructive pulmonary disease.^[3,4]^ This patient is engaged in waste recycling. His living environment is dark and humid, which is conducive to the growth of Aspergillus. Therefore, this may be the reason for his infection with invasive Aspergillus. Non-immunocompromised individuals may suffer from pulmonary aspergillosis when exposed to high levels of Aspergillus.

The clinical signs and symptoms of IPA are nonspecific and are accompanied by fever of varying intensity, cough, sputum production, dyspnea, pleuropleural pain, pleural friction, bronchospasm, and hemoptysis.^[5]^ Invasive tracheobronchial aspergillosis is a relatively rare complication.^[6]^ The main clinical manifestations of this patient were cough and fever, and no signs of bronchial invasion were found by fiberoptic bronchoscopy. There are 2 distinct types of IPA: an airway-invasive form and a vascular-invasive form, with distinct CT features.^[7]^ The angioinvasive type is characterized by hyphae penetrating the vessel wall, resulting in fungal thrombosis, followed by necrosis and hematogenous spread. This form is more common in hosts with severe neutropenia. A typical CT scan shows a large pulmonary nodule > 1 cm. There may be a halo sign around the nodule, which is distinguished by a peripheral band of ground-glass opacity that is less dense than the nodule but denser than the air in the uninvolved lung.^[8,9]^ The halo sign is an early symptom that resolves within 5 to 10 days, which reflects bleeding around the lesion.^[10,11]^ The second characteristic aspect of vascular-invasive IA is air crescent sign within the nodule. The air crescent reflects necrotic contractions, usually beginning with improvement in neutropenia.^[10]^ The chest CT of this patient was not typical at the time of admission. As the disease progressed, thin-walled cavities appeared on the chest CT. Therefore, CT scan plays a crucial role in the diagnosis and disease evolution of IPA, but it is difficult to diagnose IPA by CT alone in the early stage.

The sputum cultures of this patient were negative for many times. In addition, serum 1 to 3 β-D-Glucan (BDG) was also negative. Bronchoscopy is an important tool for the diagnosis of tracheobronchial aspergillosis. The diagnosis of this patient benefited from the pathological examination of bronchoalveolar lavage fluid. The gold standard for diagnosing IPA remains histopathology and culture.^[1]^ The pathological findings of this patient bronchoalveolar lavage fluid were consistent with Aspergillus characteristics. Aspergillus polymerase chain reaction (PCR) can be performed if necessary. The NGS of blood and sputum also showed Aspergillus in this patient. NGS is helpful for early diagnosis of IPA. In this case, the cultures of respiratory tract samples were negative for many times, so the diagnosis of IPA needs a combination of multiple methods.

Once IPA is suspected, antifungal therapy should be initiated immediately. Previous clinical studies confirmed that voriconazole improved survival and tolerability in patients with IPA.^[12,13]^ Currently, voriconazole is considered the gold standard for IPA treatment. It is recommended to monitor blood levels of therapeutic drugs to improve safety, efficacy and compliance. After 2 to 5 days of treatment, the target drug concentration for voriconazole is 1 to 5.5 mg/L.^[9]^ Amphotericin B liposomes show efficacy in second-line treatment of IPA both in children and adults.^[14,15]^ The patient was given voriconazole antifungal therapy immediately after admission, and the blood concentration of voriconazole was controlled within the target range. However, the patient treatment response was suboptimal, and amphotericin B liposome was subsequently added. Some studies also support carbofungin as a second-line treatment option, but not the first line.^[16]^ Although guidelines consistently recommend against the use of combination therapy in the first-line treatment of IA,^[17,18]^ combination drug therapy may be useful in specific settings.^[19]^ Antifungal standards recommend a minimum of 6 to 12 weeks of treatment. The exact duration of drug therapy depends on the degree of immunosuppression, responsiveness to therapy, and the site of disease.^[17]^ The patient was received the combined antifungal regimen during the treatment, but the patient did not take voriconazole regularly after discharge, resulting in the deterioration of the condition again and finally died.

In conclusion, immunocompetent adults can develop invasive pulmonary aspergillosis if they are exposed to high-risk environments. The combination of multiple methods can be helpful for the early diagnosis of IPA. If the effect of monotherapy is unsatisfactory, a combined antifungal regimen can be considered. Duration of antifungal therapy and monitoring of drug plasma concentrations are critical. IPA in non-immunocompromised host should arouse the vigilance of clinicians.

## Acknowledgments

Thanks the patient and his family.

## Author contributions

**Conceptualization:** Xiaoyun Fu.

**Data curation:** Wanping Ao, Ping Huang.

**Visualization:** Jinjing Wang.

**Writing – original draft:** Bao Fu.

**Writing – review & editing:** Bao Fu.
